# Gum Hyperplasia in a Patient with Lupus Nephritis

**DOI:** 10.34067/KID.0000000716

**Published:** 2025-07-31

**Authors:** Michelle C.Z. Chong, Henry H.L. Wu, Arvind Ponnusamy

**Affiliations:** 1 Department of Renal Medicine, Lancashire Teaching Hospitals NHS Foundation Trust, Preston, United Kingdom; 2 Renal Research, Kolling Institute of Medical Research, Royal North Shore Hospital, The University of Sydney, Sydney, New South Wales, Australia; 3 Faculty of Biology, Medicine and Health, The University of Manchester, Manchester, United Kingdom

**Keywords:** cyclosporine, immunosuppression, lupus nephritis

## Abstract

**Figure:**
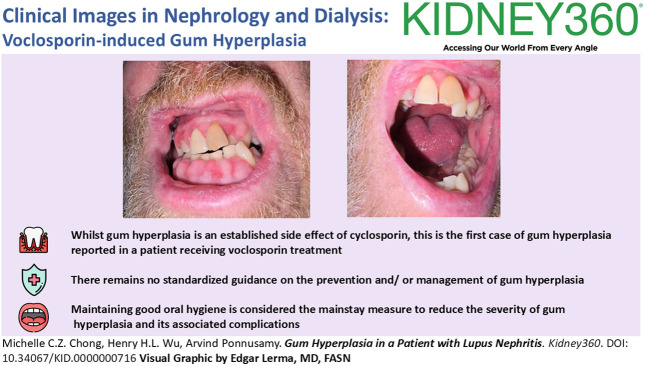

## Case Presentation

**Figure 1:**
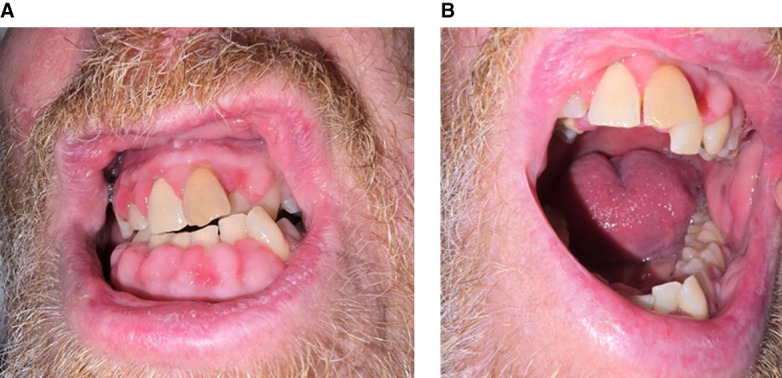
**Gum hyperplasia and gingivitis after commencement of voclosporin.** (A) Gum hyperplasia that developed 5 months after commencement of voclosporin. (B) Inflamed gums with gingival bleeding visible 7 months after commencement of voclosporin.

A 55-year-old man with cutaneous lupus in December 2022 was treated initially with hydroxychloroquine and a reducing regimen of prednisolone. The patient was referred to the nephrology clinic after nephrotic-range proteinuria (3 g/24 hours), and microscopic hematuria was observed. The patient was treated empirically with cyclophosphamide and rituximab pending kidney biopsy, which was delayed given the excess bleeding risks because of vitamin K deficiency and ongoing hypertension. Mycophenolate mofetil was commenced shortly afterward. Kidney biopsy in August 2023 revealed a diagnosis of mixed class 4 and 5 lupus nephritis (LN). He was commenced on voclosporin 23.7 mg twice daily from January 2024. Although there was improvement in the degree of proteinuria, the patient developed gum hyperplasia (Figure 1A) 5 months after commencement of voclosporin. A joint decision was made with the patient to continue with voclosporin owing to significant improvement in proteinuria. It was decided to monitor gum hyperplasia and other side effects before deciding on whether to continue with voclosporin. The patient developed gingivitis (Figure 1B) 2 months later, resulting in sepsis secondary to oral infection, which required hospitalization with intravenous antibiotics. Voclosporin was subsequently discontinued.

## Discussion

Voclosporin is a calcineurin inhibitor structurally similar to cyclosporin, the only difference being a modification at the amino acid region 1 position. This difference is believed to explain its increased affinity in binding to calcineurin, and hence its intensified immunosuppressive activity and greater treatment efficacy.^1^ The use of voclosporin in LN was brought about by the Efficacy and Safety of Voclosporin Versus Placebo for Lupus Nephritis (AURORA 1) trial,^2^ which demonstrated statistically significant positive kidney outcomes, particularly in the reduction of proteinuria. Despite its systemic potency, the cardiac and kidney-associated side effects of voclosporin have been reported to be less severe than cyclosporin. The AURORA 2 trial^3^ reinforced the safe and effective long-term use of voclosporin in LN management.

To date, voclosporin-induced gum hyperplasia has not been reported. By contrast, gum hyperplasia is an established side effect of cyclosporin, with a reported incidence ranging between 25% and 80%.^4^ Although the pathogenesis of cyclosporin-induced gum hyperplasia remains poorly understood, it is proposed that either cyclosporin or its metabolites stimulates gingival fibroblasts, where interleukin-6 secretion and synergy with interleukin-1B result in collagen synthesis and extracellular matrix formation .^5^ Increased deposition of inflammatory cells also contributes to gum hyperplasia.^5^ This process is a vicious cycle that can be aggravated by the presence of a dental plaque when oral hygiene is impaired by gum hyperplasia. Whether cyclosporin dosage correlates with the development of gum hyperplasia remains contentious. Not every individual receiving cyclosporin has gum hyperplasia, suggesting a genetic disposition may also be a factor. Perhaps a similar process resulting in voclosporin-induced gum hyperplasia also occurs. There is no standardized recommendation to prevent and/or manage gum hyperplasia. Maintaining good oral hygiene is considered the mainstay strategy of treatment to reduce the severity of gum hyperplasia. Although surgical options have been proposed, they are only indicated for cosmetic purposes.

## Teaching Points

- While gum hyperplasia is an established side effect of cyclosporin, this is the first case of gum hyperplasia reported in a patient receiving voclosporin treatment.
- There remains no standardized guidance on the prevention and/or management of gum hyperplasia. Maintaining good oral hygiene is considered the mainstay measure to reduce the severity of gum hyperplasia and its associated complications.
